# Evidence-based cardiovascular magnetic resonance cost-effectiveness calculator for the detection of significant coronary artery disease

**DOI:** 10.1186/s12968-021-00833-1

**Published:** 2022-01-06

**Authors:** Ankur Pandya, Yuan-Jui Yu, Yin Ge, Eike Nagel, Raymond Y. Kwong, Rafidah Abu Bakar, John D. Grizzard, Alexander E. Merkler, Ntobeko Ntusi, Steffen E. Petersen, Nina Rashedi, Juerg Schwitter, Joseph B. Selvanayagam, James A. White, James Carr, Subha V. Raman, Orlando P. Simonetti, Chiara Bucciarelli-Ducci, Lilia M. Sierra-Galan, Victor A. Ferrari, Mona Bhatia, Sebastian Kelle

**Affiliations:** 1 Department of Health Policy and Management, Harvard T.H. Chan School of Public Health, 718 Huntington Ave, 2nd Floor, Boston, MA 02115 USA; 2 National Taiwan University Hospital, Taipei, Taiwan; 3 Cardiovascular Division of the Department of Medicine, Brigham and Women’s Hospital, Boston, MA USA; 4 Institute for Experimental and Translational Cardiovascular Imaging, DZHK (German Centre for Cardiovascular Research) Centre for Cardiovascular Imaging, Partner Site RheinMain, University Hospital Frankfurt/Main, Frankfurt am Main, Germany; 5 Department of Cardiology, National Heart Institute, Kuala Lumpur, Malaysia; 6 Department of Radiology, Virginia Commonwealth University Medical Center, Main Hospital, Richmond, VA USA; 7 Department of Neurology, Weill Cornell Medicine/NewYork-Presbyterian Hospital, New York, NY USA; 8 Department of Medicine, University of Cape Town & Groote Schuur Hospital, Cape Town, South Africa; 9 William Harvey Research Institute, NIHR Barts Biomedical Research Centre, Queen Mary University of London, London, UK; 10 Department of Cardiovascular Medicine, Mayo Clinic, Rochester, MN USA; 11 Division of Cardiology, Cardiovascular Department, CMR Center University Hospital, Lausanne, Switzerland; 12 Faculty of Biology and Medicine, University of Lausanne, UniL, Lausanne, Switzerland; 13 Department of Medicine, School of Medicine and Public Health, Flinders University, Adelaide, Australia; 14 Department of Heart Health, South Australian Health and Medical Research Institute, Adelaide, Australia; 15 Division of Cardiology, Department of Cardiac Sciences, Stephenson Cardiac Imaging Centre, University of Calgary, Calgary, Canada; 16 Department of Radiology, Feinberg School of Medicine, Northwestern University, Chicago, IL USA; 17 Krannert Institute of Cardiology, Indiana University School of Medicine, Indianapolis, IN USA; 18 Departments of Internal Medicine and Radiology, The Ohio State University, Columbus, OH USA; 19 Royal Brompton and Harefield Hospitals, Guys’ and St Thomas NHS Hospitals and School of Biomedical Engineering & Imaging Sciences, King’s College London, London, UK; 20 Cardiovascular Division, Department of Cardiology, American British Cowdray Medical Center, Mexico City, Mexico; 21 Cardiovascular Division and Penn Cardiovascular Institute, Perelman School of Medicine, University of Pennsylvania Medical Center, Philadelphia, PA USA; 22 Department of Imaging, Fortis Escorts Heart Institute, New Delhi, India; 23 Department of Internal Medicine and Cardiology, Charité – Universitätsmedizin Berlin, Campus Virchow Klinikum, Berlin, Germany; 24 Department of Internal Medicine and Cardiology, DZHK (German Centre for Cardiovascular Research), Partner Site Berlin, German Heart Institute Berlin (DHZB), Berlin, Germany

**Keywords:** Cost-effectiveness, Cardiovascular magnetic resonance, Coronary artery disease

## Abstract

**Background:**

Although prior reports have evaluated the clinical and cost impacts of cardiovascular magnetic resonance (CMR) for low-to-intermediate-risk patients with suspected significant coronary artery disease (CAD), the cost-effectiveness of CMR compared to relevant comparators remains poorly understood. We aimed to summarize the cost-effectiveness literature on CMR for CAD and create a cost-effectiveness calculator, useable worldwide, to approximate the cost-per-quality-adjusted-life-year (QALY) of CMR and relevant comparators with context-specific patient-level and system-level inputs.

**Methods:**

We searched the Tufts Cost-Effectiveness Analysis Registry and PubMed for cost-per-QALY or cost-per-life-year-saved studies of CMR to detect significant CAD. We also developed a linear regression meta-model (CMR Cost-Effectiveness Calculator) based on a larger CMR cost-effectiveness simulation model that can approximate CMR lifetime discount cost, QALY, and cost effectiveness compared to relevant comparators [such as single-photon emission computed tomography (SPECT), coronary computed tomography angiography (CCTA)] or invasive coronary angiography.

**Results:**

CMR was cost-effective for evaluation of significant CAD (either health-improving and cost saving or having a cost-per-QALY or cost-per-life-year result lower than the cost-effectiveness threshold) versus its relevant comparator in 10 out of 15 studies, with 3 studies reporting uncertain cost effectiveness, and 2 studies showing CCTA was optimal. Our cost-effectiveness calculator showed that CCTA was not cost-effective in the US compared to CMR when the most recent publications on imaging performance were included in the model.

**Conclusions:**

Based on current world-wide evidence in the literature, CMR usually represents a cost-effective option compared to relevant comparators to assess for significant CAD.

**Supplementary Information:**

The online version contains supplementary material available at 10.1186/s12968-021-00833-1.

## Introduction

For patients with suspected coronary artery disease (CAD), cardiovascular magnetic resonance (CMR) offers a non-invasive and accurate diagnostic option. However, there are other diagnostic alternatives for such patients at low-to-intermediate risk, including ergometry, single-photon emission computed tomography (SPECT), or coronary computed tomography angiography (CCTA). Even conservative management, i.e. waiting for symptomatic disease to worsen and to manifest by complications, or more aggressive management, i.e., or immediate invasive coronary angiography (ICA), could represent options [1]. Depending on certain factors, such as imaging costs, local availability of imaging modalities and expertise and prevalence of underlying disease, tradeoffs may be realized among these options that can influence clinical decision-making [2].

Cost-effectiveness analysis can be used to quantitatively weigh tradeoffs between length of life, quality of life, and incurred costs, across different diagnostic strategies, allowing payers and physician decision-makers to choose a higher-value pathways [3, 4]. Most health technology assessments in high-income countries use cost-per-quality-adjusted life-year (QALY) to quantify value from cost-effectiveness analyses, with estimated costs including both immediate imaging costs and all downstream costs (including both additional care stemming from test results and saved costs from averted coronary heart disease events, follow-up tests, and procedures) [5]. Although there have been prior publications evaluating the clinical and cost impacts of CMR-based diagnostic strategies in patients with suspected CAD, there has been less clarity on the specific cost-effectiveness profile of CMR compared to its relevant comparators across both clinical settings and patient types, particularly as evidence of imaging performance continues to evolve [6–8].

In a 2-step approach, we first analyzed and summarized the comparative cost effectiveness surrounding use of CMR imaging for the assessment of patients presenting with stable chest pain syndromes compared to its relevant comparators based on the existing medical literature. Then we created a unique cost-effectiveness calculator that could be used globally to estimate lifetime discounted costs and QALYs for CMR versus its relevant comparator techniques. To facilitate global use for different international geographic regions or referral populations, adjustment of context-specific patient and system-level inputs (such as disease prevalence and imaging costs) was incorporated into the cost-effectiveness calculator.

## Methods

### Search strategy of existing literature and data extraction

We conducted a systematic literature review using the Tufts Cost-Effectiveness Analysis Registry (CEA Registry, www.cearegistry.org) and PubMed for English-language cost-effectiveness published from 2005 to 2020 [9, 10]. The CEA Registry contains 8000 English-language cost-per-QALY studies. The CEA Registry uses keywords such as QALYs, quality adjusted, and cost-utility analysis to search PubMed for English-language publications. The reference lists of every identified CMR cost-per-QALY or relevant review study were searched to identify additional CMR cost-effectiveness studies missed by our other methods. We only included studies that used CMR as an imaging strategy to assess for CAD as the primary clinical condition. Two reviewers (YY and AP) independently reviewed each study to extract relevant data, and resolved any differences in data extractions at in-person meetings; a third author (SK) was contacted when consensus could not be reached at the in-person meetings. Detailed information on the search strategy and data extraction for the CEA Registry is reported elsewhere [10, 11].

The search terms used to identify cost-per-QALY studies were combinations of methodological terms (cost-effectiveness, QALY, incremental cost-effective ratio) and clinical terms (cardiac magnetic resonance, CMR, coronary angiography). Articles other than cost-effectiveness studies that were related to the overall value of CMR (reviews, meta-analyses, diagnostic performance, editorials, etc.) were identified but not included in our systematic review (Appendix Fig. 3). We reviewed the cost-effectiveness studies that included CMR as a strategy and summarized the following information among these articles: setting of the analysis, comparators included, analytic perspective taken, analytic time horizon taken, main conclusion on the cost effectiveness of CMR (where “cost-effective” was defined as either health-improving and cost saving [“dominant”] [3] or having a cost-per-QALY or cost-per-life-year result lower than the cost-effectiveness threshold [4]), and key drivers of the results. We performed a sensitivity analysis excluding papers that reported cost-effectiveness outcomes other than cost-per-QALY or cost-per-life year (such as cost-per-case detected).

We used author assessments to determine whether CMR represented a cost-effective option or cost-ineffective option for a given paper, which could depend on country-specific cost-effectiveness thresholds. In the United States (US), for example, the American College of Cardiology (ACC) and the American Heart Association (AHA) issued a joint statement on health care “value” in 2014 that specified that cost-per-QALY results below $50,000/QALY indicate high-value care, cost-per-QALY estimates between $50,000/QALY and $150,000/QALY indicate intermediate-value care, and cost-per-QALY estimates greater than $150,000/QALY indicate low-value care [12]. We also categorized some papers as showing unclear CMR cost-effectiveness when there was considerable uncertainty around the CMR cost-effectiveness results.

### Development of the CMR cost effectiveness calculator (meta-model)

We built a user-friendly CMR cost effectiveness meta-model based on a larger CMR Markov (state-transition cohort) model developed for a prior CMR cost-per-QALY study (by study co-authors AP, YG, RK) performed for a US health care system perspective [13]. The model projects lifetime discounted QALY and cost outputs for five strategies: (1) no imaging; (2) CMR; (3) SPECT; (4) CCTA; (5) ICA. Other strategies, such as echocardiography, were not included in the larger Markov model. In this model, patients in the CMR, SPECT, and CCTA groups underwent ICA only if noninvasive imaging demonstrated abnormal findings. Those with positive ICA results (with some having also received FFR) were assumed to undergo both medical and revascularization therapies, and this combination led to overall improved health outcomes (quantified using lifetime discounted QALYs). Patients with normal findings were presumed to be free of obstructive CAD and were managed accordingly. In the no imaging strategy patients were initially managed without any investigations. Assuming escalating symptoms in 58% of patients with obstructive CAD who did not receive treatment in their first year after assessment (i.e., patients with false negative results) would return within the first year and undergo ICA, leading to medical and revascularization therapies (i.e., 58% of false negatives would experience the same outcomes as true positives within 1 year) [14]. The Markov model had four major health states: no clinical major cardiovascular events [MACE, defined as: cardiovascular death, acute nonfatal myocardial infarction (MI), hospitalization for unstable angina or heart failure], history of one MACE, history of more than one MACE, and all-cause death [13]. In the Markov model, QALYs are estimated by combining the length of life patients spend in each of the health states with a quality-of-life value (“utility”) ranging between 0 (representing death) and 1 (perfect health), with no MACE having an average utility value of 0.84 and MACE having an average utility of 0.78 [15]. Life costs estimated by the Markov model depend on the cost of the imaging strategies used, procedures performed, and the acute and long-term healthcare-related costs associated with each health state (with MACE states having 1st-year costs between $11,000–18,000 and subsequent annual costs of $3400) [13]. Both QALY and cost outcomes can be discounted at an annual rate (such as 3%, recommended for cost-effectiveness analyses performed for the US setting) [16].

The linear regression-based meta-model was trained (i.e., coefficients were estimated) on 100,000 model input–output combinations from the probabilistic sensitivity analysis (2nd-order Monte Carlo simulation) from the larger Markov simulation model [17]. Previous studies have shown that simple linear regression-based meta-models can approximate larger disease models with high accuracy (r-squared values that often exceed 0.95). The meta-model allows users to replicate the larger Markov model results and change 10–20 key meta-model inputs to view corresponding meta-model outputs to create customized results for specific scenarios or populations of interest [18, 19]. Meta-model variable selection was based on a 0.05 level of significance for beta coefficients in the linear regressions. We validated our meta-model on a separate 1000 model input–output combinations from the probabilistic sensitivity analysis (2nd-order Monte Carlo simulation) from the larger Markov simulation model that were not used to train the meta-model (i.e., the test set) [17]. Meta-model goodness-of-fit was assessed using adjusted r-squared and percentage deviation (for external validation) metrics for the test set.

The meta-model approximates the lifetime discounted QALY and cost results for a given imaging strategy based on user-entered population- and system-level inputs, such as prevalence of CAD, costs of imaging and procedures, and other model inputs listed in Appendix Table 3. We used the meta-model to explain observed differences in conclusions from published cost-effectiveness analyses included in our literature review that were performed in the US setting. Specifically, we used our meta-model to replicate the cost-effectiveness results of the US study it was based on Ge et al. [13], which found CMR to be cost-effective compared to CCTA, SPECT, ICA, and no imaging strategies [13]. Then we changed key input parameters of the meta-model (such as imaging performance and cost parameters, CAD prevalence, treatment costs, etc.) to replicate the cost-effectiveness results of another US study (with a similar model structure and decision problem) that found CCTA to be cost-effective compared to CMR [20]. Using the meta-model, we then changed only the imaging performance inputs from the values in Genders et al. [20] cost-effectiveness study to the more recent imaging performance inputs used by Ge et al. [13] to determine whether these imaging performance inputs alone could explain the difference in CMR cost-effectiveness results.

The larger Markov model was programmed in TreeAge Pro 2019 software (version 19.2.1; TreeAge Software, LLC, Williamstown, Massachusetts, USA), the meta-model was created using RStudio (version 1.2.5042; RStudio Software, Boston, Massachusetts, USA), and the user-friendly cost-effectiveness calculator is programmed in Microsoft Excel (Microsoft Corporation, Redmond, Washington, USA) and is included as a downloadable spreadsheet in Additional file 1 and as a web-based tool at: https://docs.google.com/spreadsheets/d/12TMgbIS6sDpXkafSYfNaNw8vSx-LCTCE4K_yAa7mdyY/edit?usp=sharing.

## Results

Our search yielded 39 studies of which 15 ultimately met our inclusion criteria (shown in Table 1 and Appendix Fig. 3) [13, 14, 20–30]. We excluded 17 studies because they were not original research (such as perspective articles or literature reviews), they focused on the cost-effectiveness of CMR for heart failure patients (as opposed to CAD), or they did not include CMR as a comparator. Of the remaining studies, an additional seven were excluded for not being a cost-effectiveness analysis (such as studies evaluating only costs or clinical effectiveness). Further two studies were excluded in our sensitivity analysis restricting our analysis to cost-per-QALY or cost-per-life-year studies [24, 29].

**Table 1:** Summary of the cost-effectiveness literature of CMR for CAD (15 total studies)

Attribute	# of studies	% of studies (%)	Study citations
Setting			
US setting	5	33	Moschetti et al. [24], Stojanovic et al. [29], Sharples et al. [28], Genders et al. [20], Ge et al. [13, 31]
European setting (including the UK)	11	73	Walker et al. [14], Boldt et al. [22], Thom et al. [30], Petrov et al. [25], Pontone et al. [27], Pletscher et al. [26], Moschetti et al. [24], Sharples et al. [28], Genders et al. [20], Campbell et al. [23], Walker et al. [31]
Other setting	2	13	Bertoldi et al. [21], Kozor et al. [32]
Comparators included			
No imaging	2	13	Genders et al. [20], Ge et al. [13]
Stress echocardiography	4	27	Thom et al. [30], Sharples et al. [28], Genders et al. [20], Bertoldi et al. [21]
Stress electrocardiography	4	27	Walker et al. [14], Pletscher et al. [26], Bertoldi et al. [21], Walker et al. [31]
SPECT	11	73	Walker et al. [14], Boldt et al. [22], Thom et al. [30], Pletscher et al. [26], Sharples et al. [28], Genders et al. [20], Stojanovic et al. [29], Sharples et al. [28], Ge et al. [13], Walker et al. [31], Kozor et al. [32], Walker et al. [31]
CCTA	5	33	Pontone et al. [27], Genders et al. [20], Bertoldi et al. [21], Ge et al. [13]
Immediate coronary angiography	10	67	Walker et al. [14], Boldt et al. [22], Thom et al. [30], Petrov et al. [25], Pletscher et al. [26], Moschetti et al. [24], Sharples et al. [28], Ge et al. [13], Walker et al. [31], Kozor et al. [32]
Main conclusion on CMR value			
CMR cost-effective	10	67	Walker et al. [14], Boldt et al. [22], Petrov et al. [25], Pontone et al. [27], Pletscher et al. [26], Moschetti et al. [24], Stojanovic et al. [29], Ge et al. [13], Walker et al. [31], Kozor et al. [32]
CMR not cost-effective	2	13	Genders et al. [20], Bertoldi et al. [21]
Unclear cost-effectiveness	3	20	Thom et al. [30], Sharples et al. [28], Campbell et al. [23]
Key drivers of cost-effectiveness results			
Underlying CAD prevalence	9	60	Walker et al. [14], Boldt et al. [22], Thom et al. [30], Pletscher et al. [26], Moschetti et al. [24], Stojanovic et al. [29], Genders et al. [20], Ge et al. [13], Kozor et al. [32]
Test costs	4	27	Pletscher et al. [26], Moschetti et al. [24], Sharples et al. [28], Bertoldi et al. [21]
Perspective taken			
Societal	1	8	Genders et al. [20]
Healthcare system or payer	10	67	Walker et al. [14], Boldt et al. [22], Pletscher et al. [26], Moschetti et al. [24], Stojanovic et al. [29], Bertoldi et al. [21], Campbell et al. [23], Ge et al. [13], Walker et al. [31], Kozor et al. [32]
Hospital	1	7	Stojanovic et al. [29]
Not clearly stated	4	27	Thom et al. [30], Petrov et al. [25], Pontone et al. [27], Sharples et al. [28]
Model time horizon			
Lifetime	7	47	Walker et al. [14], Pletscher et al. [26], Genders et al. [20], Bertoldi et al. [21], Campbell et al. [23], Ge et al. [13], Kozor et al. [32]
10–30 years	2	13	Boldt et al. [22], Petrov et al. [25]
3–10 years	1	7	Thom et al. [30]
< 3 years	4	27	Pontone et al. [27], Stojanovic et al. [29], Sharples et al. [28], Walker et al. [31]
Not stated	1	7	Moschetti et al. [24]

### Summary of methods used for CMR cost-effectiveness studies

Table 1 shows that most of the 15 studies that met our inclusion criteria were performed in a US (five studies) [13, 20, 24, 28, 29] or European setting (seven included the United Kingdom [14, 20, 23, 24, 28, 30, 31], three included Germany [22, 24, 25]). One study (Bertoldi et al.) was performed for the Brazilian public health system [21] and another (Kozer et al.) was performed for the Australian health care system [32]. In 11 of the 15 studies CMR was compared to SPECT strategies, in 10/15 immediate ICA, in 5/15 to CCTA, and 4/15 to stress electrocardiography. Simulation models (such as decision trees or state transition models) were used for 10/15 studies [13, 14, 20–24, 26, 29, 32], and seven of these ten studies extrapolated outcomes for a lifetime time horizon; the five studies not using simulation models relied on empirical data [25, 27, 28, 30, 31] resulting in time horizons of less than 10 years.

### Summary of results of CMR cost-effectiveness studies

CMR was found to be cost-effective versus its relevant comparator in 10/15 studies [13, 14, 22, 24–27, 29, 31, 32]. Among these ten studies, the most common comparators to CMR were strategies that used ICA [13, 14, 22, 24–26, 31, 32] or SPECT [13, 14, 22, 26, 29, 31, 32], while three studies compared CMR versus strategies that used CCTA [13, 27, 31], and one directly compared CMR to a no imaging strategy [13].

Two studies concluded that CCTA was more cost-effective (Bertoldi et al. and Genders et al.) compared to CMR [20, 21]. In sensitivity analyses, Bertoldi et al. found that a cost reduction of 79% was required for the CMR strategy to be cost-effective versus the CCTA from the Brazilian public health system perspective [21]. This result was driven by only slightly higher QALYs from the CMR strategy compared to the CCTA strategy based on sensitivity and specificity inputs from meta analyses published in 2010 [33] (for CMR) and 2008 [34] (for CCTA). Genders et al. focused on a low-to-moderate risk population (performing separate analyses for men and women) and found that CCTA as a first-line test (combined with baseline echocardiography in all patients and additional invasive diagnostic work-up in patients with positive CCTA) almost always dominated CMR in the three settings analyzed (the United States, the Netherlands, and the United Kingdom) [20]. These results were primarily driven by assuming superior accuracy for CCTA (sensitivity of 0.98 and specificity of 0.89) [34–36] compared to CMR (0.89 and 0.76, respectively) [37]. Model-based cost-effectiveness studies by Walker et al. and Ge et al. used similar model structures as applied by Genders et al., but with CMR operating characteristics based on more recent trials and meta-analyses [7, 8, 38]. With this approach CMR was cost-effective compared to other imaging strategies such as ICA [13, 14], SPECT [13, 14], and CCTA (with CT-derived fractional flow reserve) [13].

Three studies found unclear cost-effectiveness among the imaging strategies analyzed [23, 28, 30], including two studies that were cost-effectiveness analyses conducted alongside a randomized controlled trial, which were not powered to show statistically significant differences in cost-effectiveness outcomes [28, 30]. Campbell et al. developed a simulation model that included positron emission tomography (PET) for the United Kingdom health care payer perspective and found in probabilistic sensitivity analyses that there were similar probabilities of CMR or PET being optimal in the relevant cost-effectiveness threshold ranges for the United Kingdom [23].

Across the 15 studies included in our review, nine found that underlying prevalence of CAD was a key driver of the cost-effectiveness findings [13, 14, 20, 22, 24, 26, 29, 30, 32], and four found that these results were sensitive to changes in imaging prices [21, 24, 26, 28]. In our sensitivity analysis restricting inclusion to cost-per-QALY or cost-per-life-year studies, 8/13 found CMR to be cost-effective [13, 14, 22, 25–27, 31, 32], 3/13 showed uncertain cost-effectiveness rankings [23, 28, 30], and 2/13 concluded that CCTA was superior [20, 21]. We did not find evidence that CMR was more or less likely to be cost-effective across settings; among the 5 studies that were performed for the US setting [13, 20, 24, 28, 29], 3 found that CMR was cost-effective [13, 24, 29], which was similar to the proportion of all studies that found CMR to be cost-effective (10 out of 15).

### CMR cost effectiveness calculator (meta-model) results

The fitted coefficients for the lifetime discounted QALY and cost results for ‘No Imaging’ strategy, and incremental QALY and cost results for the ‘CMR’, ‘CCTA’, ‘SPECT’, and ‘Immediate ICA’ strategies are shown in Appendix Table 3. Validation using the test sets showed good fits for each model, with r-squared values ranging from 0.846 to 0.999 across the ten meta-models, with percentage deviation varying from − 0.117% to 0.028%, which are also shown in Appendix Table 3. Table 2 shows the cost-effectiveness analysis results of our meta-model compared to: (1) the model originally used to derive it (i.e., a replication of the Ge et al. cost-effectiveness results using our meta-model); (2) the Genders et al. study that concluded that CCTA was more cost-effective than CMR in the US (i.e., a replication of the Genders et al. cost-effectiveness results using our meta-model); (3) and meta-model results using key input values from the Genders et al. paper (such as CMR and CCTA sensitivity and specificity, CMR and CCTA costs, underlying prevalence of CAD, and other selected inputs) with Ge et al. model inputs for all other inputs (see Appendix Table 4 for full list) [13, 20].

**Table 2:** Published and meta-model lifetime discount quality-adjusted life year, costs, and cost-effectiveness results for selected imaging strategies and scenarios

Strategy	Costs	QALYs	ICER
Published Ge et al. [13] results			
No imaging	$16,936	12.721	Reference
CMR	$19,273	12.765	$52,000/QALY
CCTA	$19,886	12.765	Dominated by CMR
Meta-model with Ge et al. [13] inputs^A^ (replication of Ge et al. [13])			
No imaging	$19,223	12.160	Reference
CMR	$21,962	12.204	$63,000/QALY
CCTA	$22,578	12.203	Dominated by CMR
Published Genders et al. [20] results for men			
No imaging	$6827	11.062	Reference
CCTA	$13,177	11.840	$29,000/QALY
CMR	$14,172	11.840	Dominated by CCTA
Published Genders et al. [20] results for women			
No imaging	$7506	12.110	Reference
CCTA	$14,109	12.340	$29,000/QALY
CMR	$15,198	12.330	Dominated by CCTA
Meta-model with selected Genders et al. [20] inputs^B^ (replication of Genders et al. [20])			
No imaging	$12,549	12.844	Reference
CCTA	$14,487	12.889	$42,000/QALY
CMR	$14,289	12.884	Dominated by CCTA
Meta-model with selected Genders et al. [20] inputs except Ge et al. sensitivity/specificity^C^			
No imaging	$12,549	12.844	Reference
CMR	$13,997	12.885	$34,000/QALY
CCTA	$14,823	12.885	Dominated by CMR

^A^CMR sensitivity = 0.89, CMR specificity = 0.87, CMR cost = $807, CCTA sensitivity = 0.90, CCTA specificity = 0.71, CCTA cost (includes FFR for some patients) = $981, age = 62.5 years, coronary angiography cost = $3941, CABG cost = $38,979, PCI cost = $36,556, prevalence of coronary artery disease = 32.4%, proportion male = 53%

^B^CMR sensitivity = 0.89, CMR specificity = 0.76, CMR cost = $621, CCTA sensitivity = 0.98, CCTA specificity = 0.89, CCTA cost = $372, age = 60 years, coronary angiograph cost = $2989, CABG cost = $38,217, PCI cost = $6529, prevalence of coronary artery disease = 30.0

^C^CMR sensitivity = 0.89, CMR specificity = 0.87, CMR cost = $621, CCTA sensitivity = 0.90, CCTA specificity = 0.71, CCTA cost = $372, age = 60 years, coronary angiograph cost = $2989, CABG cost = $38,217, PCI cost = $6529, prevalence of coronary artery disease = 30.0

**Fig. 1:**
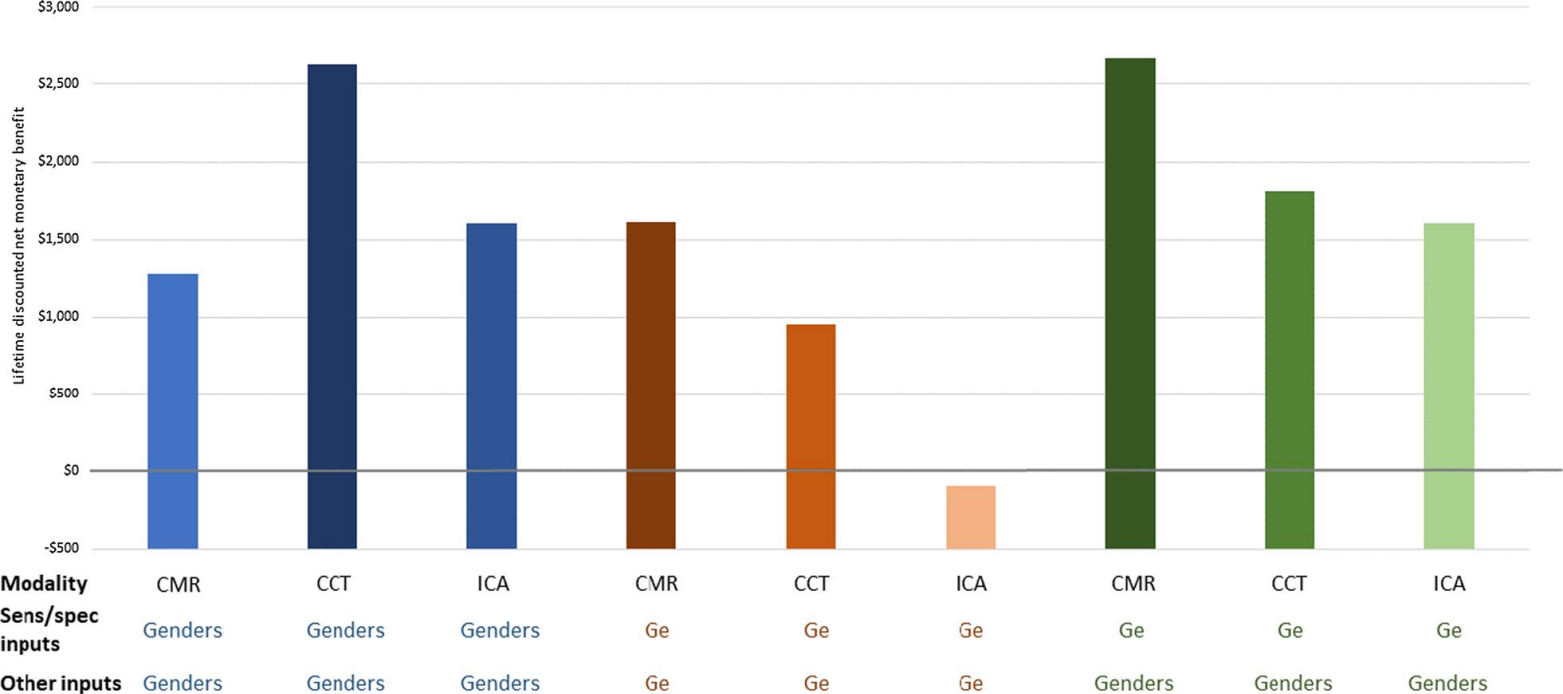
Meta-model lifetime discounted incremental net monetary benefit results (compared to ‘No Imaging’); higher incremental net monetary benefit indicates better cost effectiveness profile. Net Monetary Benefit (NMB) is a single metric that monetizes QALYs (using willingness-to-pay of $100,000/QALY) and subtracts costs. *CMR* cardiovascular magnetic resonance, *CCT* coronary computed tomography angiography, *ICA* invasive coronary angiography; Genders et al. [20]; Ge et al. [13]

**Fig. 2:**
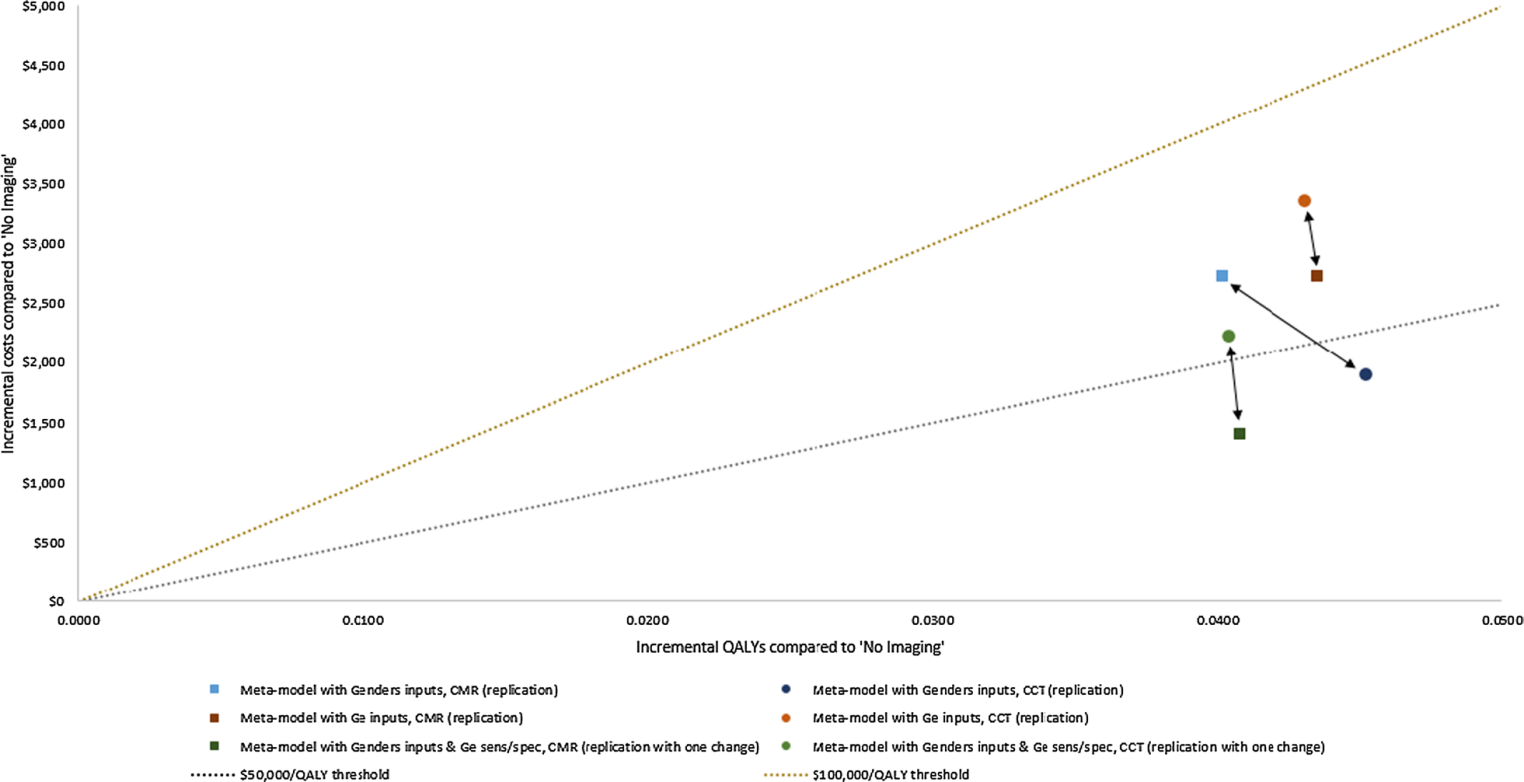
Meta-model lifetime discounted quality-adjusted life year and cost results (compared to ‘No Imaging’). Squares indicate CMR, circles indicate CCT, colors indicate meta-model input sources, arrows represent comparisons of CMR vs. CCT for a given inputs source (strategies to the bottom and right to their comparators have higher quality adjusted life years (QALYs) and lower costs, i.e. they are dominant strategies), dotted lines represent cost-effectiveness thresholds (strategies below cost-effectiveness thresholds are good value compared to ‘No Imaging’)

We were able to use the meta-model to closely replicate the incremental cost-effectiveness results for CCTA compared to a ‘No Imaging’ strategy and to CMR (Table 2); when the Genders et al. model inputs values were used, CCTA dominated CMR (i.e. CCTA had greater QALYs and lower costs) [20]. When the Ge et al. model input values were used, CMR dominated CCTA, replicating the cost-effectiveness comparisons from the Ge et al. analysis [13, 20]. When the meta-model was fed with Genders et al. inputs except for the updated sensitivity and specificity values as used by Ge et al., CMR dominated CCTA (Table 2, Figs. 1 and 2). CMR would have to cost 182% of our base-case estimate ($807 to $1465) before the CCTA strategy would be considered more cost-effective (using a willingness-to-pay of $100,000 per QALY). Appendix Table 5 shows other threshold values for imaging performance, prevalence, and age inputs for CMR and the other imaging strategies we evaluated using the meta-model.

## Discussion

Our systematic literature review found that most (62%) studies formally evaluating the cost-effectiveness of CMR compared to other relevant imaging options for patients with suspected CAD world-wide concluded that:

- CMR-based diagnostic strategies produced health at reasonable value compared to setting-specific cost-effectiveness thresholds.
- When CMR is not available, CCTA represents a cost-effective alternative compared to a no imaging strategy or immediate ICA strategy, which is consistent with current recommendations of major international cardiac societies [39].

These cost-effectiveness outcomes depend on the operating characteristics of the imaging modality of interest and the underlying prevalence of CAD in the population of interest. It is therefore of importance for readers of such cost-effectiveness analyses to scrutinize the sources of these key variables when interpreting the resulting cost-effectiveness outcomes. Publications which concluded that CCTA dominated CMR used inputs in the cost-effectiveness models which were outside the range of the majority of reported results. We also found that relying on a single data source to estimate the cost-effectiveness of CMR, such as a cost-effectiveness analysis conducted alongside a clinical trial, could lead to uncertain results (e.g. when such trials are underpowered for cost and QALY outcomes).

Considering uncertain and setting-specific inputs that could drive cost-effectiveness results, we developed a CMR cost-effectiveness calculator that end-users (physicians, hospital decision-makers, guidelines writers, payers, health economist researchers) can use to approximate setting-specific cost-effectiveness results. The ability to iteratively generate these estimates is crucial given the importance of weighting potential tradeoffs between length of life, quality of life, and local costs across imaging modalities available to physicians aiming to diagnosis and treat ischemic heart disease, and the setting-specific and evolving nature of these inputs. The calculator also allows to determine strategies, which allow to achieve diagnosis at the lowest possible cost, or to treat significant CAD with the lowest cumulative cost of care. As expected, changing the sensitivity and specificity of CMR or CCTA changes the relative cost effectiveness of each of these modalities. Our cost-effectiveness calculator allows users to update these inputs as newer evidence and meta-analyses are published. Our replication and adaptation of the Genders et al. study showed this explicitly. Other key drivers of CMR cost-effectiveness, such as disease prevalence and imaging or treatment costs, can also be setting-specific, and we found there are many settings (almost all non-US/non-European countries, with the exceptions of one study for Brazil and one study for Australia) without any formal cost-effectiveness analyses published. Users can use our tool world-wide to better align their local understanding of model inputs to cost-effectiveness results.

Our study focused on cost-per-QALY or cost-per-life-year-saved studies, but there are other types of economic evaluation studies beyond formal cost-effectiveness analyses (such as cost-minimization analyses) that we identified comparing CMR to other imaging strategies used to diagnose CAD [40]. Data from the European CMR registry [41], which contains data from 59 medical centers across 18 countries, were used for two such analyses that compared CMR to ICA-based strategies; these studies both found that the CMR-based strategy would result in cost savings compared to inpatient ICA, driven by the costs differences between strategies and reduced revascularization procedures in the CMR-based strategies [42, 43]. A randomized controlled trial assigning 109 patients to either a CMR observation unit arm or usual inpatient care arm found that the CMR-based care arm reduced cardiac-related costs during the hospitalization and over the first year post-discharge [44]. These studies were not included in our literature review as they did not include a QALY or life-year effectiveness measure, but they add to our overall study conclusion that CMR can represent high- or intermediate-value care, or even produce cost-savings, depending on the imaging strategy CMR is being compared to. Future clinical studies providing sex-specific inputs (on imaging performance, for instance) could also help reveal whether the cost-effectiveness of CMR differs for men and women.

## Limitations

Our study has limitations that should be noted. For our systematic literature review, we were limited to the existing published literature. There is publication bias in terms of what settings cost-effectiveness of CMR studies are performed for (with US, Germany, and the United Kingdom relatively overrepresented), and there is the possibility of financial or non-financial bias in the study authors that could affect which model inputs they choose (thus affecting model results). Our cost-effectiveness calculator tool can somewhat mitigate this bias, if users have non-biased inputs they believe would better reflect the current state of the evidence or their local populations or settings. The cost-effectiveness calculator is based on a meta-model of a larger Markov model, and therefore the calculator contains many model limitations of the larger model in addition to its own imperfect ability to replicate the model results. To overcome this limitation, we performed a model validation analysis on the meta-model using data that were specifically not used to generate the model. Our meta-model was also limited to the strategies evaluated in the larger Markov model [13], which is why echocardiography is not included as a comparator in the meta-model. Due to data limitations, we also did not model potential side effects from contrast agents used for CCTA or CMR or radiation exposure from CCTA, which would have amplified our lifetime discount QALY meta-model results comparing CCTA to CMR [45]. Finally, our review and cost calculator focus on cost-effectiveness outcomes, which might not capture all dimension that are relevant for health policy or clinical decision-making, missing elements such as equity and patients’ preference considerations.

Despite those limitations, cost-effectiveness analysis represents the field’s best attempt to systematically and fairly quantify the value of health care interventions, a notion supported by the 2014 ACC-AHA policy statement that uses cost-effectiveness metrics to differentiate high-value care (health gains are worth their costs) from low-value care (prices should be lowered to be in line with the health gains that are produced by the intervention of interest) [12].

## Conclusions

The majority of cost-effectiveness evidence evaluating CMR-based diagnostic strategies for patients with suspected CAD identify CMR to be a cost-effective imaging strategy, delivering high-value care in many settings. When comparing diagnostic techniques, the optimal strategy depends on factors that change over time and across clinical settings, such as imaging performance, imaging costs, and disease prevalence. Therefore, decision-makers should contextualize existing literature with additional information, whether from their local data when available or through use of a tool like the cost-effectiveness calculator we present here. Such tools can assist in obtaining the most realistic estimates of overall value from options available to diagnose and treat significant CAD.

### Supplementary Information

**Additional file 1.** Users can enter model inputs that relate to their population of interest (e.g., age, probability of patient having treatable CAD), imaging strategies (sensitivity, specificity, cost), treatments (e.g., effectiveness, risks, and costs of revascularization procedures), and other variables (e.g., willingness-to-pay for health, discount rate), to then see how these collection of inputs translates to lifetime per-person net monetary benefit (i.e., an overall metric of value using cost-effectiveness analysis), lifetime discounted quality-adjusted life years (i.e., the main measure of effectiveness), and lifetime discounted costs. Default input values for the US are provided in the model file.

## Figures and Tables

**Fig. 3:**
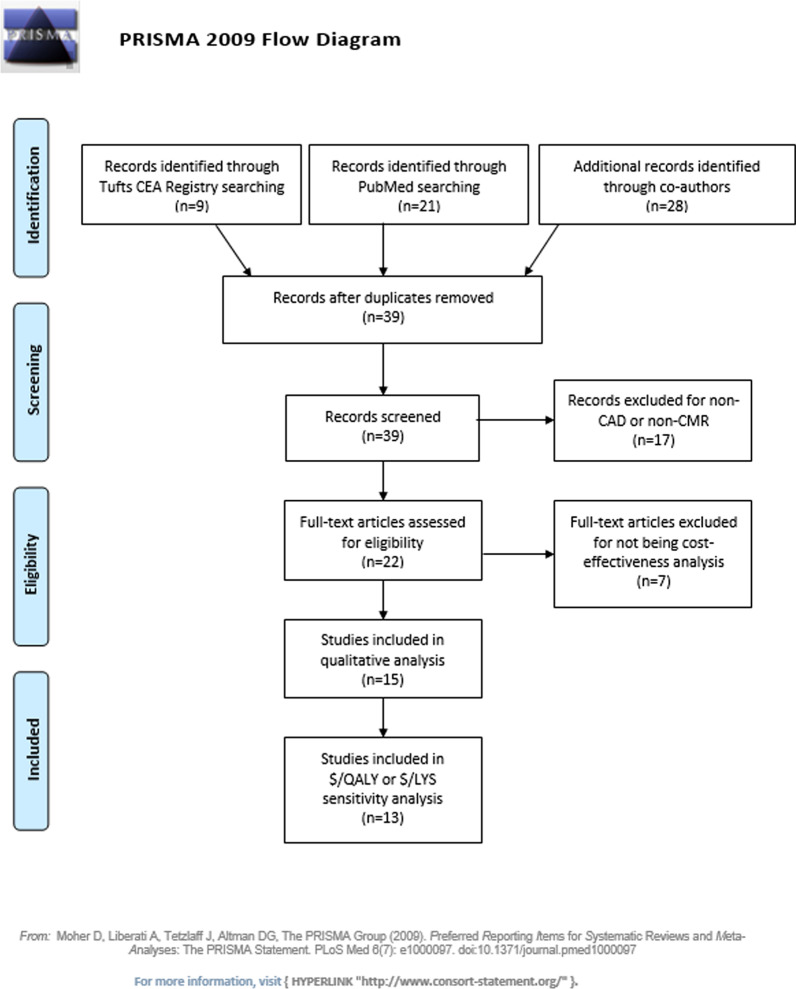
Search strategy and paper selection process

## Data Availability

The data used to calculate all meta-model results is contained in the “CMR CEA Calculator_Demo” Excel file, which is included as a Additional file for this paper and at: https://docs.google.com/spreadsheets/d/12TMgbIS6sDpXkafSYfNaNw8vSx-LCTCE4K_yAa7mdyY/edit?usp=sharing.

## Appendix

See Fig. 3, Tables 3, 4, 5.

**Table 3:** Meta-model inputs, regression coefficients, and performance metrics

Variable	Meta-model coefficient						
	No imaging QALY	No imaging cost	Incremental QALY CMR, SPECT, CCTA^a^	Incremental cost CMR, SPECT^a^	Incremental cost CCTA^b^	Incremental QALY immediate ICA^a^	Incremental cost immediate ICA^a^
Intercept	32.5468	− 1124.0687	− 0.0108	− 930.0	− 1257.9	0.0246	− 832.2
Age in years	− 0.2343	− 239.1547	− 0.0002	12.8481	13.0193	− 0.0001	14.6191
Probability of patient having CAD	− 4.0808	50,086.6	0.1366	4335.6	3826.9	n/a	1643.7
Sensitivity of CMR	n/a	n/a	0.0484	1749.6	n/a	n/a	n/a
Specificity of CMR	n/a	n/a	0.0055	− 2649.1	n/a	n/a	n/a
Sensitivity of CCTA	n/a	n/a	n/a	n/a	1797.7	n/a	n/a
Specificity of CCTA	n/a	n/a	n/a	n/a	− 2655.3	n/a	n/a
Probability patient with false negative result returns to get coronary angiography within 1 year	0.0958	4034.8	− 0.0963	− 3562.4	− 3595.5	− 0.1082	− 4014.4
Annual rate of having a new MACE event for patients without CAD	− 75.8	287,992.6	n/a	n/a	n/a	0.0768	n/a
Post-1st year rate of having a MACE event for patients with CAD who received revascularization procedure	− 13.8625	29,804.1	0.2165	16,366.8	16,579.2	0.2434	18,378.7
Hazard rate ratio for patients who received medical therapy and revascularization procedure (natural log)	0.2203	528.3	− 0.1499	− 577.3	− 587.7	− 0.1685	− 645.2
Probability of dying during PCI	n/a	n/a	− 0.2742	n/a	n/a	− 0.3074	n/a
Probability of dying during CABG	n/a	n/a	− 0.1148	n/a	n/a	− 0.1263	n/a
Cost of CMR (or SPECT)	n/a	n/a	n/a	0.9987	n/a	n/a	n/a
Cost of CCTA	n/a	n/a	n/a	n/a	1.000	n/a	n/a
Cost of FFR-CT (added to CCTA for some patients)	n/a	n/a	n/a	n/a	0.4068	n/a	n/a
Cost of immediate coronary angiography with FFR	n/a	n/a	n/a	0.0534	0.0982	n/a	0.2967
Cost of immediate coronary angiography without FFR	n/a	n/a	n/a	0.0775	0.1416	n/a	0.4279
Cost of CABG	n/a	0.0851	n/a	0.0131	0.0133	n/a	0.0147
Cost of PCI	n/a	0.1901	n/a	0.0294	0.0300	n/a	0.0331
Acute (1st-year) cost of non-fatal MACE	n/a	0.1817	n/a	− 0.0034	− 0.0038	n/a	− 0.0045
Acute (1st-year) cost of fatal MACE	n/a	0.1233	n/a	− 0.0024	− 0.0021	n/a	− 0.0032
Utility of coronary heart disease health state	1.0242	n/a	− 0.0277	n/a	n/a	− 0.0312	n/a
Annual discount rate for costs and QALYs	− 126.8	− 82,131.3	− 0.0108	5653.8	5623.4	− 0.4461	6349.3

Meta model performance	No imaging QALY	No imaging Cost	Incremental QALY CMR^b^	Incremental cost CMR^c^	Incremental cost CCTA^a^	Incremental QALY immediate ICA^a^	Incremental cost immediate ICA^a^
External validation r-squared	0.9757	0.9133	0.9757	0.9982	0.9861	0.9999	0.9858
External validation deviation compared to Markov model	0.046%	− 0.025%	− 0.048% to 0.064%	− 0.031% to − 0.018%	0.007%	− 0.001%	− 0.040%

n/a = not included (not beta coefficient not significant at an alpha = 0.05 level)

^a^Incremental compared to ‘No Imaging’

^b^Incremental compared to ‘No Imaging’; the same coefficients were used for incremental CMR, CTTA, and SPECT QALYs, given the exact same model structure and type of model inputs

^c^Incremental compared to ‘No Imaging’; this meta-model (costs for CCTA) had different model inputs due to some patients receiving FFR (not the case for CMR or SPECT costs)

**Table 4:** Comparison of selected model inputs from Ge et al. [13] and Genders et al. [20]

Variable	Genders 2015 value [20]	Genders source(s)	Ge 2020 value [13]	Ge source(s)
CMR sensitivity	0.89	Jaarsma et al. [37]	0.89	Knuuti et al. [8]
CMR specificity	0.76	Jaarsma et al. [37]	0.87	Knuuti et al. [8]
CMR cost	$621	CPT 75563 and 93015	$807	CMS
CCTA sensitivity	0.98	Mowatt et al. [34], Scheutz et al. [36], von Ballmoos et al. [35]	0.90	Danad et al. [7]
CCTA specificity	0.89	Mowatt et al. [34], Scheutz et al. [36], von Ballmoos et al. [35]	0.71	Danad et al. [7]
CCTA cost (includes FFR for some patients)	$372	CPT 75574	$981	CMS
Age (years)	60	Assumption	62.5	Kwong et al. [46]
ICA cost	$2989	CPT 93454	$3941	CMS
CABG cost	$38,217	AHRQ 2011	$38,797	O’Sullivan et al. [47]
PCI cost	$6529	CPT 92980	$36,556	O’Sullivan et al. [47]
Prevalence of CAD	0.30	Nieman et al. [48]	32.4%	Kwong et al. [46]
Proportion male	Sex-specific analyses	Not applicable	53%	Kwong et al. [46]

*AHRQ* Agency for Healthcare Research and Quality, *CABG* coronary artery bypass grafting, *CAD* coronary artery disease, *CCTA* coronary computed tomographic angiography, *CMR* cardiovascular magnetic resonance, *CPT* current procedural terminology, *ICA* invasive coronary angiography, *FFR* fractional flow reserve, *ICER* incremental cost-effectiveness ratio, *MACE* major adverse cardiovascular event(s), *MI* myocardial infarction, *PCI* percutaneous coronary intervention, *QALY* quality-adjusted life year, *SPECT* single-photon emission computed tomography, *XCA* x-ray coronary angiography

**Table 5:** Threshold values for selected meta-model inputs where the net monetary benefit (i.e., overall value quantified as monetized QALYs at $100,000/QALY minus costs) of the CMR strategy is equal to a competing strategy (Ge 2020 value represents base-case model input value)

Variable	Base-case values in meta-model		Calculated threshold values to be equivalent to CMR strategy			
	Ge 2020 value [13]	Ge source(s)	No imaging^a^	CCTA^a^	SPECT^a^	XCA^a^
CMR sensitivity	0.89	Knuuti et al. [8]	0.37 (42%)	0.68 (76%)	0.55 (62%)	0.34 (38%)
CMR specificity	0.87	Knuuti et al. [8]	0.37 (43%)	0.66 (76%)	0.55 (63%)	0.34 (39%)
CMR cost	$807	CMS	$2420 (300%)	$1465 (182%)	$1840 (228%)	$2522 (313%)
Age (years)	62.5	Kwong et al. [46]	None	None	None	None
Prevalence of CAD	32.4%	Kwong et al. [46]	15% (47%)	None	None	66% (204%)

*CAD* coronary artery disease, *CCTA* coronary computed tomographic angiography, *CMR* cardiovascular magnetic resonance, *SPECT* single-photon emission computed tomography, *QALY* quality-adjusted life year, *XCA* x-ray coronary angiography

^a^Threshold value for this model input parameter that sets net monetary benefit (i.e., value) of CMR strategy equal to this strategy. “None” indicates not threshold value result exists for this parameter (i.e., CMR strategy is optimal over full range of possible values holding all other model inputs constant). Parentheses indicate percent of base-case value used be Ge et al. [13]

## Abbreviations

ACC: American College of Cardiology
AHA: American Heart Association
AHRQ: Agency for Healthcare Research and Quality
CABG: Coronary artery bypass grafting
CAD: Coronary artery disease
CCTA: Coronary computed tomographic angiography
CMR: Cardiovascular magnetic resonance
CPT: Current procedural terminology
ESC: European Society of Cardiology
FFR: Fractional flow reserve
ICA: Invasive coronary angiography
ICER: Incremental cost-effectiveness ratio
MACE: Major adverse cardiovascular event(s)
MI: Myocardial infarction
PCI: Percutaneous coronary intervention
PET: Positron emission tomography
QALY: Quality-adjusted life year
SPECT: Single-photon emission computed tomography
XCA: X-ray coronary angiography
